# Linear Quadratic Regulator Control of Vehicle Active Front Steering Considering Aerodynamic Characteristics

**DOI:** 10.3390/s26134140

**Published:** 2026-07-01

**Authors:** Junzhi Hu, Conghao Liu, Yunlong Wang, Yilong Sun, Liang Hao

**Affiliations:** 1 School of Automobile and Traffic Engineering, Liaoning University of Technology, Jinzhou 121001, China; 2 School of Mechanical Engineering and Automation, Northeastern University, Shenyang 110819, China

**Keywords:** active front steering, sensor feedback, crosswind, handling stability, linear quadratic regulator control

## Abstract

**Highlights:**

**What are the main findings?**

- The effectiveness of the AFS controller based on the LQR algorithm was validated.
- Under continuous sinusoidal steering, the AFS controller actively regulated the special vehicle.

**What are the implications of the main findings?**

- Using the LQR algorithm, the special vehicle achieved improved steering performance and a stable body attitude under crosswind disturbances.
- The LQR algorithm improves the handling stability and driving safety of the special vehicle.

**Abstract:**

This study enhanced the handling stability and driving safety of a special vehicle by developing a vehicle dynamics model using TruckSim 2019. An ideal two-degree-of-freedom vehicle model was established using Simulink. The reference yaw rate and vehicle sideslip angle were derived from the reference model. Fluent simulations were performed on the vehicle to obtain the aerodynamic coefficients as functions of the relative inflow angle. These relationships were fitted to functional expressions and integrated into the aerodynamic module of TruckSim, replacing the default coefficient curves and improving the accuracy of the subsequent simulations. To improve steering performance, an active front steering (AFS) controller based on the linear quadratic regulator (LQR) algorithm was designed, and an AFS control strategy based on sensor feedback was implemented using MATLAB/Simulink 2021b. Finally, simulations were performed to validate the effectiveness of the controller, which showed that under continuous sinusoidal steering, the controller regulated the vehicle. By applying a front-wheel steering angle computed using the LQR algorithm, the actual yaw rate and vehicle sideslip angle closely tracked the reference values. Using the LQR algorithm, the vehicle achieved improved steering performance and a stable body attitude under crosswinds. Thus, the LQR algorithm enhanced the handling stability and driving safety of the vehicle.

## 1. Introduction

With the rapid proliferation of automobiles, driver assistance and intelligent driving technologies have rapidly advanced. Active front steering (AFS) systems designed to improve vehicle steering performance have entered mass production. Conventional mechanical steering systems have a fixed steering ratio, which limits their ability to handle complex driving environments. Moreover, they exhibit heavy steering efforts at low speeds and poor controllability at high speeds. An AFS system overcomes these limitations and has significant advantages [1].

Owing to the increasing number of traffic accidents caused by external disturbances and human error, vehicle driving safety has become a critical bottleneck hindering the advancement of road transportation. The vehicle handling stability depends heavily on the steering system. Therefore, to improve both driving safety and handling stability, in-depth investigations on AFS technology have been conducted. Mammar and Baghdassarian [2] established the control objectives and steering system architecture for AFS to regulate the vehicle yaw rate and further investigated AFS control strategies under various driving scenarios. The simulation results indicated that the proposed control algorithm is both reasonably designed and effective, leading to noticeable improvements in steering performance.

To enhance the maneuverability and stability of articulated vehicles, Kim et al. [3] designed an active steering controller based on the linear quadratic regulator (LQR) theory. They conducted low- and high-speed simulation tests using a TruckSim–Simulink co-simulation platform. The results showed that the active steering controller significantly improves maneuverability at low speeds and lateral stability at high speeds. Wang et al. [4] proposed an integrated active steering control strategy for articulated vehicles, to ensure safe driving. Using this control strategy, the low-speed maneuverability and high-speed stability of an articulated vehicle were validated through simulations. The results confirmed that the integrated active steering system significantly improves both the lateral stability and path-tracking performance of the articulated vehicle. Deng et al. [5] designed an LQR-based active rear-steering controller to enhance the trajectory tracking accuracy of intelligent vehicles under extreme operating conditions. A TruckSim–MATLAB/Simulink co-simulation platform was developed to evaluate the characteristics of the designed controller. The simulation results indicated that combining LQR active steering with a linear time-varying model predictive control (LTVMPC) yaw moment control strategy substantially reduces the vehicle sideslip angle and yaw rate during lane-change maneuvers. This combination effectively improves the lateral stability of articulated heavy vehicles.

A review of the existing literature shows that most studies on AFS control focus on passenger cars, whereas research on special vehicles remains limited. Most of these studies were conducted under normal driving conditions, and investigations of crosswind disturbances during driving remain insufficient. Recent studies have also explored advanced vehicle stability control methods, including MPC-based control [6], robust control, and integrated steering and yaw-moment control strategies [7]. In addition, vehicle sideslip angle is often obtained through observer-based estimation methods rather than direct measurement in practical applications [8]. However, studies focusing on special vehicles subjected to crosswind-induced aerodynamic disturbances remain limited. Compared with the above-mentioned studies on AFS-LQR control for heavy vehicles, the present work distinguishes itself in three aspects. First, those existing studies mainly focused on articulated vehicles or passenger cars, whereas this study targets a special vehicle with a unique aerodynamic profile, for which crosswind sensitivity is more pronounced. Second, unlike previous works that rely on default empirical aerodynamic coefficients in TruckSim, we replace them with coefficient functions fitted from Fluent simulations as a function of the relative inflow angle. Third, the proposed LQR controller explicitly uses sensor feedback to compare real vehicle states with reference values from an ideal 2-DOF model. These features collectively provide a more realistic evaluation of AFS performance under crosswind disturbances for a special vehicle than previous LQR-based studies.

**Figure 1:**
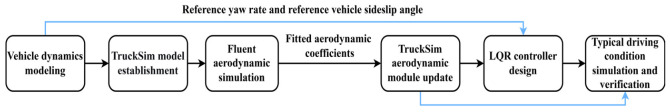
Overall research flowchart.

Modern active front steering systems rely on vehicle-state feedback to achieve accurate steering control and handling stability improvement. In practical vehicle applications, yaw rate sensors, steering angle sensors, wheel speed sensors, and inertial measurement units (IMUs) are commonly used to obtain real-time vehicle dynamic states for feedback control [9]. Under crosswind disturbances, accurate state sensing is particularly important because aerodynamic interference may significantly affect vehicle lateral stability. Therefore, integrating sensor-feedback information with the LQR-based AFS control strategy is beneficial for improving vehicle handling stability and driving safety. The overall research flowchart is shown in Figure 1.

## 2. Vehicle Dynamics Model

### 2.1. Ideal Vehicle Dynamics Model

**Figure 2:**
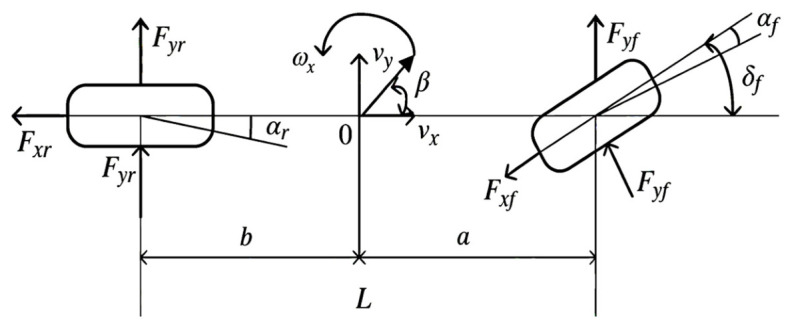
Linear 2-DOF vehicle model.

Simplifying the vehicle model effectively reduced the computational burden and improved the efficiency of the controller system. By simplifying the four wheels to single front and rear wheels, a two-degree-of-freedom (2-DOF) vehicle model was obtained [10]. This model fully captures the lateral and yaw motions of a vehicle while driving without requiring a complete full-vehicle model. The specific model is shown in Figure 2.

The following assumptions were made for this model to better investigate the problem:

(1) The system input was the front-wheel steering angle, and the steering system was disregarded.
(2) The vehicle moved only parallel to the ground; therefore, the suspension system was ignored.
(3) The tire cornering characteristics remained within the linear region. The tire sideslip angle was positively correlated with the lateral force, and the aligning torque effect was neglected.
(4) Disturbances from air resistance and rolling resistance during driving were not considered.
(5) The vehicle speed remained constant during operation.

Based on these assumptions, 2-DOF dynamic equations were derived using Newton’s second law. Here, u denotes the constant forward speed of the vehicle, v denotes the lateral speed, β = v/u is the vehicle sideslip, and $\omega_{r}$ is the yaw rate.

The equations of motion for the system can be expressed as

(1) $$\begin{array}{ll} \sum F_{y} & =m\left(\dot{v}+u\omega_{r}\right)\\ \sum M_{z} & =I{\dot{\omega }}_{r} \end{array},$$

where $F_{yf}$ denotes the total lateral force on the front axle and $F_{yr}$ denotes that on the rear axle. After simplification and rearrangement, the differential equations of motion for the 2-DOF vehicle were derived as

(2) $$m\left(\dot{v}+u\omega_{r}\right)=k_{1}\delta_{f}-\left(k_{1}+k_{2}\right)\beta -\frac{ak_{1}-bk_{2}}{u}\omega_{r},$$

(3) $$I_{z}{\dot{\omega }}_{r}=ak_{1}\delta_{f}-\left(ak_{1}-bk_{2}\right)\beta -\frac{\left(a^{2}k_{1}+b^{2}k_{2}\right)}{u}\omega_{r},$$

where u is the vehicle speed; $K_{1}\;and\;K_{2}$ are the cornering stiffnesses of the front and rear axles, respectively; a and b are the distances from the center of mass to the front and rear axles, respectively; $\delta_{f}$ is the front wheel steering angle; m is the vehicle mass; and $I_{z}$ is the moment of inertia about the z-axis.

In steady-state driving, both the yaw rate and the vehicle sideslip angle remain constant, yielding $\dot{\beta }=0$ and ${\dot{\omega }}_{r}=0$. However, when a vehicle travels on low-adhesion road surfaces or tires operate in a nonlinear region, these conditions no longer hold. In such cases, the lateral acceleration $a_{y}$ of the vehicle can be expressed as

(4) $$a_{y}=u\omega_{r}+a_{x}\tan\beta +\frac{u}{\sqrt{1+\tan^{2}\beta }}\dot{\beta },$$

where $\dot{\beta }$ denotes the time derivative of the vehicle sideslip angle.

At high vehicle speeds, the vehicle sideslip angle becomes negligible. The lateral acceleration can be approximated as

(5) $$a_{y}\approx u\omega_{r}<\mu g.$$

In Equation (5), μ denotes the tire friction coefficient and g is the gravitational acceleration.

By summarizing the above derivation, the upper bound of the yaw rate was obtained. The reference yaw rate and reference vehicle sideslip angle can be expressed as

(6) $$\begin{array}{ll} \beta_{d} & =\frac{b+u^{2}\frac{am}{2Lk_{1}}}{1+ku^{2}}\cdot \frac{\delta_{f}}{L}\\ \omega_{rd} & =\frac{u}{1+ku^{2}}\cdot \frac{\delta_{f}}{L} \end{array}.$$

Considering the constraint imposed by the tire–road friction limit, the upper bounds of the reference values are as follows:

(7) $$\begin{array}{ll} \beta_{d,\max\;} & =\arctan(0.02\mu g)\\ \omega_{rd,\max\;} & =0.85\frac{\mu g}{u} \end{array},$$

where $k=\frac{m}{L^{2}}\left(\frac{a}{k_{2}}-\frac{b}{k_{1}}\right)$ and L denotes the wheelbase.

According to the tire–road friction constraint, the maximum achievable lateral acceleration is limited by $a_{y}<\mu g$. Under steady-state cornering and constant-speed conditions, the lateral acceleration can be approximated as $a_{y}\approx u\omega_{r}$, leading to the yaw rate limit $\omega_{r}\leq \frac{\mu g}{u}$. To provide an additional stability margin and avoid operation near the friction limit, a factor of 0.85 is adopted in this study.

The vehicle dynamics are expressed in state-space form. The state equation of the linear two-degree-of-freedom (2-DOF) vehicle model is given by:

(8) $$\dot{X}=Ax+Bu,$$

In the above equation, $X={\left[\beta \;\omega_{r}\right]}^{T}$ denotes the state variable and $U=\delta_{f}$ represents the input.

Specifically, it is expressed as:

(9) $$\begin{array}{ll} \dot{X} & =A\left[\begin{array}{c} \beta \\ \omega_{r} \end{array}\right]+B\delta_{f}\\ Y & =\left[\begin{array}{c} a_{y}\\ \omega_{r} \end{array}\right]=C\left[\begin{array}{c} \beta \\ \omega_{r} \end{array}\right]+D\delta_{f} \end{array},$$

where $A=\left[\begin{array}{cc} a_{11} & a_{12}\\ a_{21} & a_{22} \end{array}\right]=\left[\begin{array}{cc} \frac{k_{1}+k_{2}}{mv} & \frac{ak_{1}-bk_{2}}{mv^{2}}-1\\ \frac{ak_{1}-bk_{2}}{I_{z}} & \frac{a^{2}k_{1}+b^{2}k_{2}}{I_{z}v} \end{array}\right]$;$\;B=\left[\begin{array}{c} b_{11}\\ b_{12} \end{array}\right]=\left[\begin{array}{c} -\frac{k_{1}}{mv}\\ -\frac{ak_{1}}{I_{z}} \end{array}\right]$; $C=\left[\begin{array}{cc} c_{11} & c_{12}\\ c_{21} & c_{22} \end{array}\right]=\left[\begin{array}{cc} \frac{k_{1}+k_{2}}{m} & \frac{ak_{1}-bk_{2}}{mv}\\ 0 & 1 \end{array}\right]$;$\;D=\left[\begin{array}{c} d_{11}\\ d_{12} \end{array}\right]=\left[\begin{array}{c} \frac{k_{1}}{m}\\ 0 \end{array}\right]$.

The initial state of the system is given by: $X={\left[0\;0\right]}^{T}$.

For the ideal 2-DOF model, the vehicle speed and front-wheel steering angle serve as inputs, and the system outputs two response variables: the reference vehicle sideslip angle and the reference yaw rate. To distinguish these from the actual yaw rate $\omega_{r}$ and vehicle sideslip angle β during real driving, the output values derived from the ideal 2-DOF model are defined as the reference yaw rate $\omega_{rd}$ and the reference vehicle sideslip angle $\beta_{d}$. These values were used as reference targets for the subsequent AFS controller when regulating the vehicle steering.

It should be noted that the ideal 2-DOF vehicle model adopted in this study is based on several simplifying assumptions, including linear tire behavior, constant longitudinal velocity, and the neglect of suspension dynamics and aerodynamic disturbances. Therefore, the model is primarily intended to generate the reference yaw rate and reference vehicle sideslip angle for controller design rather than to fully reproduce the complete vehicle dynamics under all operating conditions. Although strong crosswinds may introduce additional aerodynamic nonlinearities that are not explicitly represented in the ideal 2-DOF model, these effects are captured by the TruckSim vehicle model through the aerodynamic coefficient functions obtained from Fluent simulations. Consequently, the reference model can still provide reasonable target states for the AFS controller while the full-vehicle model accounts for the complex aerodynamic influences. For the moderate lateral dynamic responses investigated in this study, the ideal 2-DOF model provides an effective and widely accepted approximation for vehicle stability control design.

### 2.2. TruckSim Model Establishment

A full-vehicle model of a special vehicle was developed using TruckSim software, incorporating suspension, braking, and tire models, and other subsystems. Subsequently, the road model and external crosswind environment required for the simulation tests were configured to establish the simulation environment in advance for validating the AFS controller algorithm. Table 1 lists the overall dimensions and the fundamental structural parameters of the special vehicle.

This special vehicle is a two-axle heavy-duty military modular cabin vehicle with a relatively high center of gravity (ground clearance of 1250 mm) and a total curb weight of 7800 kg. The front track width is 2030 mm. The distance from the center of mass to the front and rear axles is 1400 mm and 1250 mm, respectively, with a wheelbase of 2650 mm. The front axle is a steering axle with a maximum load capacity of 7 tons, and the rear axle is a drive axle with a rated load of 15.5 tons. The vehicle is equipped with a diesel engine (rated power 300 kW, speed 2000 rpm), an 8-speed manual transmission, and an open differential with a transmission ratio of 4.1. The main parameters of tires are shown in the Table 2.

The road model was designed as a combined straight and curved road in accordance with the test road specified in GB/T 41796-2022 [11]. The first 4 s of driving corresponds to a straight section. From 4–20 s, the vehicle travelled through a curved section with a curvature that was defined as required. This study adopted a combination of fixed and gradually varying curvature [12]. Specifically, the fixed-curvature segment had a radius of 400 m. The transition segment connecting the straight section and fixed-curvature curve was configured with a gradually increasing curvature. Along this transition, the curvature increased from 0 to 0.003 $m^{-1}$, and the curvature change rate did not exceed $4\times 10^{-5}{\;m}^{-2}$. In TruckSim, the external crosswind disturbances acting on the vehicle was realized through the built-in fan model, and the crosswind was specifically simulated by varying its parameters.

### 2.3. Sensor Feedback Architecture

The proposed AFS controller operates based on vehicle-state feedback. In practical vehicle control systems, yaw rate sensors, steering angle sensors, wheel speed sensors, and inertial measurement units (IMUs) are commonly employed to obtain real-time vehicle dynamic states. In this study, the reference yaw rate and reference vehicle sideslip angle were generated using the ideal 2-DOF vehicle model, whereas the actual vehicle states used for feedback control were obtained from the corresponding TruckSim vehicle-state outputs.

The sensed vehicle states were compared with the reference values generated by the ideal vehicle model, and the LQR controller computed an additional front-wheel steering angle to improve vehicle handling stability under crosswind disturbances.

## 3. Aerodynamic Analysis of the Special Vehicle

The default aerodynamic coefficients in the traditional TruckSim are typically based on static or simplified dynamic models. By contrast, functions fitted from the Fluent (Ansys Workbench 2020r2) data can capture more complex dynamic characteristics. These fitted functions accurately represent the relationship between the aerodynamic coefficients, yaw rate, and lateral speed during crosswind simulations [13]. Moreover, the aerodynamic coefficients computed using Fluent account for the actual vehicle body shape and complex flow field effects, making them more accurate than the simplified default model in TruckSim. Particularly, under crosswind conditions, Fluent captures complex phenomena, such as flow separation and vortex formation [14]. By fitting these high-accuracy results to functional expressions, they can be converted into parameters that can be used directly in TruckSim. This allows the calculated lateral displacement, lateral force, and yaw moment to closely match the actual physical conditions.

Seamless coupling between Fluent and TruckSim is fundamentally enabled by their shared physical foundations. Both the fluid computations in Fluent and the aerodynamic forces acting on the vehicle in TruckSim are physically described by the Navier–Stokes equations [15]:

(10) $$\rho \left(\frac{\partial \vec{V}}{\partial t}+\vec{V}\cdot \nabla \vec{V}\right)=-\nabla p+\nabla \cdot \tau +\vec{f},$$

where ρ denotes the fluid density, $\vec{V}$ is the velocity vector field, $\frac{\partial \vec{V}}{\partial t}$ represents the local acceleration, $\vec{V}\cdot \nabla \vec{V}$ is the convective acceleration, −∇p is the pressure gradient force, ∇⋅τ is the divergence of the viscous stress tensor, and $\vec{f}$ denotes the body force.

In Fluent, the air surrounding the vehicle is discretized into a computational mesh using the finite-volume method. The software directly solves the Reynolds-averaged Navier–Stokes (RANS) equations and continuity equation [16]. TruckSim does not solve these partial differential equations directly. However, the aerodynamic force formulations used in TruckSim are essentially the result of integrating the Navier–Stokes equations over the vehicle surface. The total aerodynamic force ${\vec{F}}_{aero}$ acting on the vehicle body is the sum of the surface pressure and viscous forces:

(11) $${\vec{F}}_{aero}=\int_{S}\left(-p\vec{n}+\tau \cdot \vec{t}\right)dS,$$

where p andτ are the precise physical quantities obtained by Fluent when solving the Navier–Stokes equations.

Furthermore, in both Fluent post-processing and TruckSim parameter settings, the aerodynamic coefficients ($C_{d},C_{s},C_{l}$) are defined by identical formulas [17]:

(12) $$C_{F}=\frac{F_{actual}}{q\cdot A_{ref}}=\frac{F_{actual}}{\frac{1}{2}\rho v^{2}A_{ref}}.$$

To ensure the reliability of the aerodynamic coefficients, some CFD settings were used in Fluent. The computational domain is a rectangular box, measuring 160 m (length) × 45 m (width) × 20 m (height), with a blockage ratio of about 5% according to SAE J1252 standards. The vehicle model was simplified by removing small items like mirrors and wipers, but the overall body shape was retained. For the mesh, tetrahedral meshes were used overall, and multilayer designs were applied for the boundary layer. The mesh skewness was set with a maximum of 0.89 and an average of 0.23, which ensures good usability. After these settings were done, a mesh quality evaluation was performed, and the mesh quality satisfies the requirements of the present study. The turbulence model chosen was the realizable k−ε model, because when applied to boundary layer flows with high adverse pressure gradients, it has a shorter simulation time and accurate results. For the boundary conditions: first, the crosswind inlet was specified as a velocity inlet boundary condition, and the outlet boundary was specified as a pressure outlet. For the vehicle itself, the positive direction of the vehicle speed $V_{x}$ is along −x, and for the wind speed $V_{y}$, −y is defined as the positive direction. Additionally, a separate resultant speed $V_{s}$ is set as the relative speed of the study object in a side wind environment. For the ground and the top, according to the needs of this study, a stationary ground with no-slip condition is used.

Through a flow field analysis of the research vehicle, the trends of the aerodynamic coefficients as functions of the relative inflow angle were obtained. To understand this relationship better, the data points were fitted to a functional curve using mathematical methods. A comparison with the findings of other studies [18,19] shows that the aerodynamic coefficients generally exhibit a consistent trend as the relative inflow angle varies.

**Figure 3:**
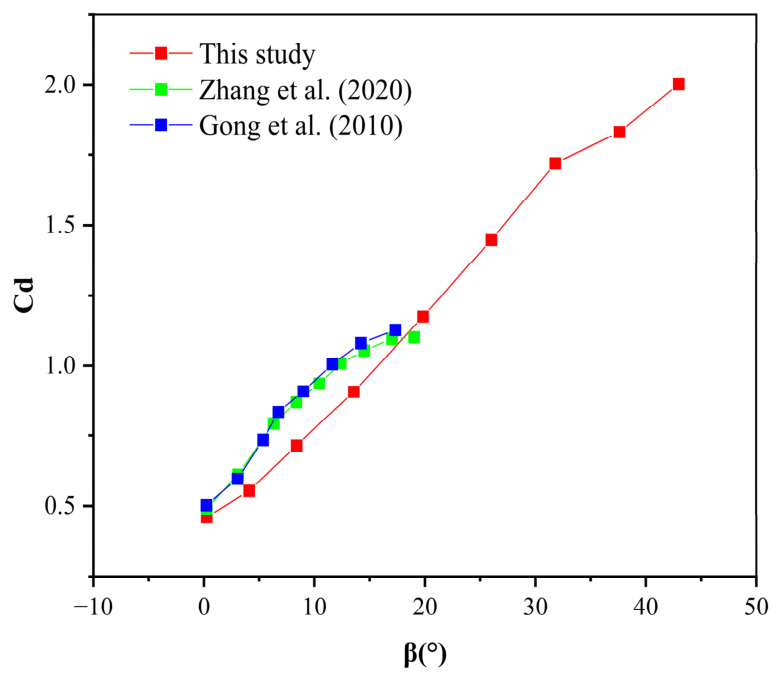
Variation in drag coefficient with relative inflow angle [18,19].

**Figure 4:**
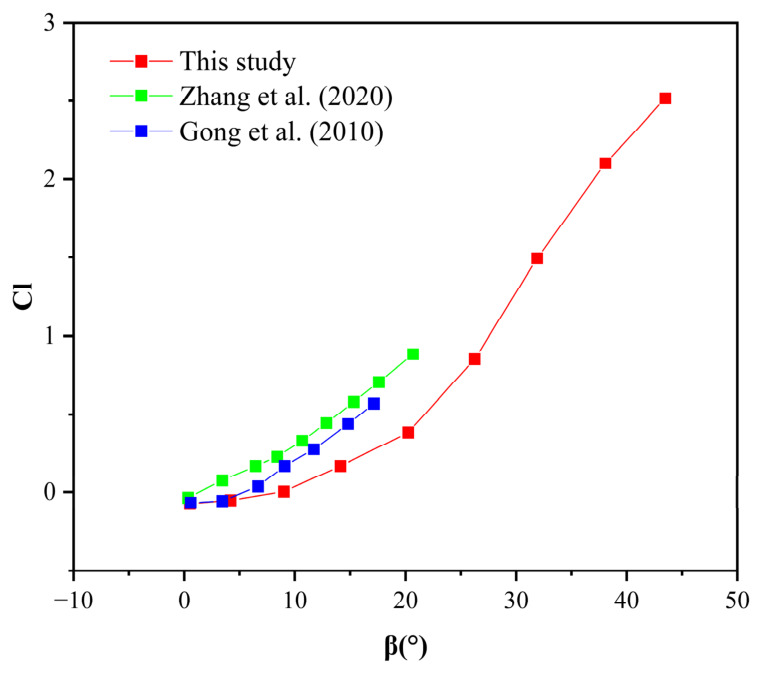
Variation in lift coefficient with relative inflow angle [18,19].

**Figure 5:**
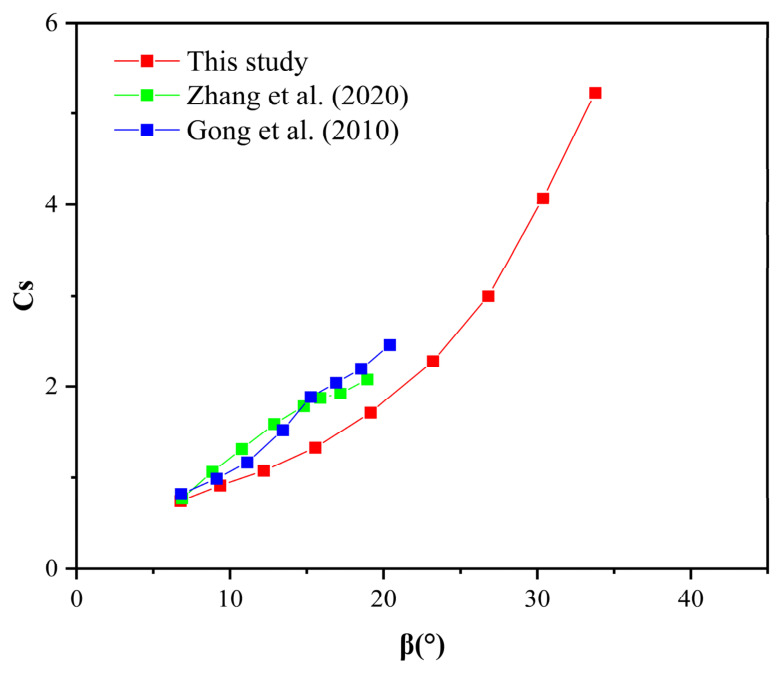
Variation in side-force coefficient with relative inflow angle [18,19].

**Figure 6:**
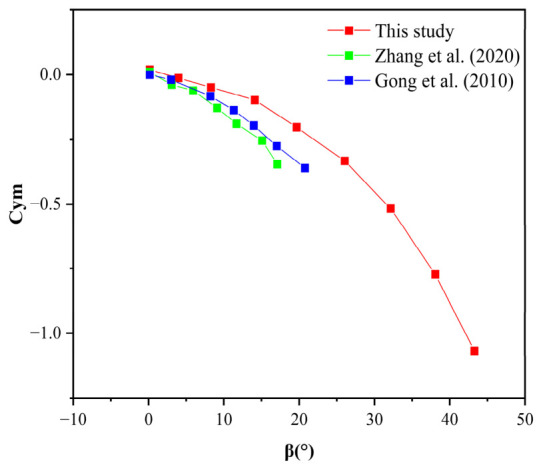
Variation in yaw-moment coefficient with relative inflow angle [18,19].

The simulation results for the aerodynamic coefficients are presented in Figure 3, Figure 4, Figure 5 and Figure 6, which show how each coefficient varies with the relative inflow angle. A comparison of the four coefficients revealed that the side force coefficient exhibited the fastest variation and steepest curve slope. As the relative inflow angle increased, the yaw moment coefficient also increased progressively, following a trend largely consistent with the simulation results of other studies. Overall, the observed variations in the aerodynamic coefficients with relative inflow angle were reasonable.

**Figure 7:**
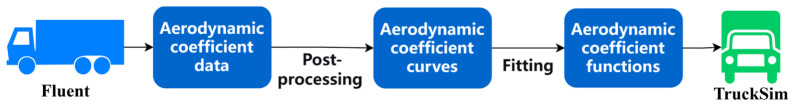
One-way coupling flowchart.

When analyzing the aerodynamic characteristics of a vehicle in Fluent, a fixed wind speed is usually set, and a simulation is run to obtain the results. However, this approach does not adequately capture the continuously varying crosswinds experienced by a vehicle during actual driving. To address this limitation, the data were first fitted to functional expressions using the MATLAB Curve Fitting Toolbox and then imported into TruckSim. This method yielded results that are more consistent with real-world conditions and, therefore, are more accurate. The workflow is illustrated in Figure 7.

**Figure 8:**
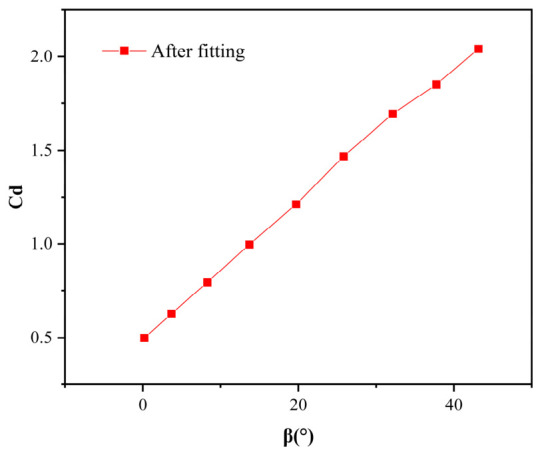
Drag coefficient fitting.

**Figure 9:**
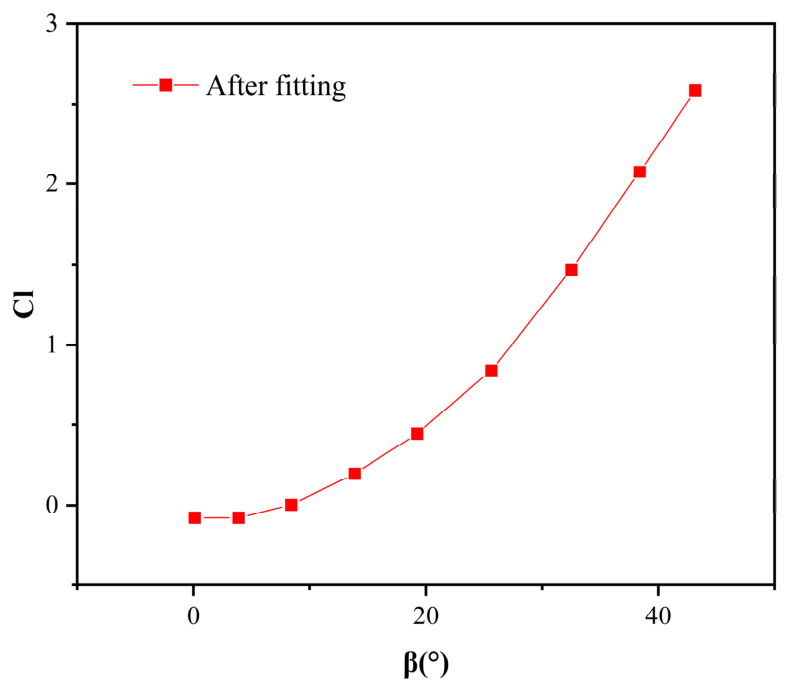
Lift coefficient fitting.

**Figure 10:**
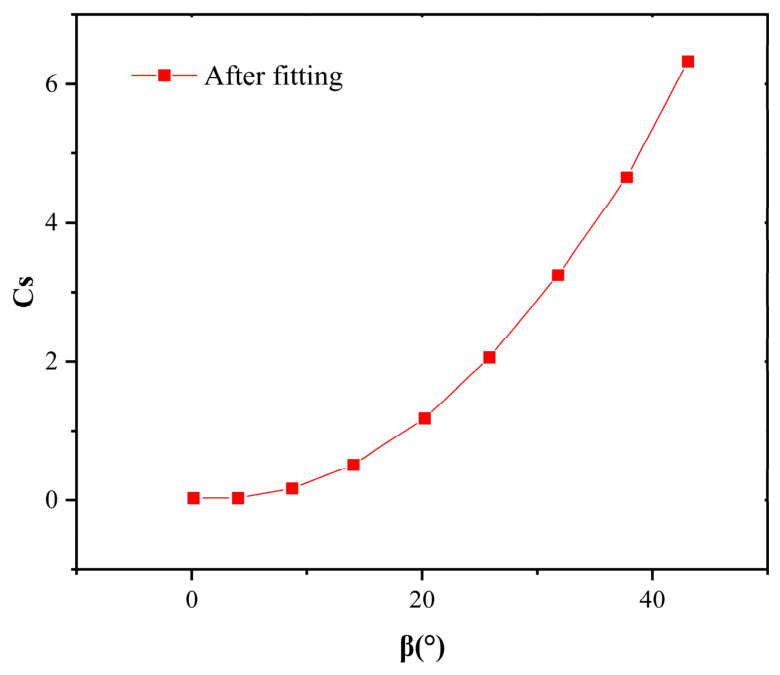
Side force coefficient fitting.

**Figure 11:**
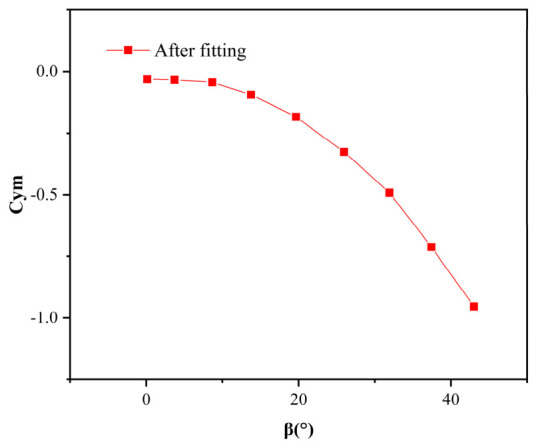
Yaw moment coefficient fitting.

Figure 8, Figure 9, Figure 10 and Figure 11 present the fitted function curves for each aerodynamic coefficient. To quantitatively evaluate the reliability of the fitted aerodynamic coefficients, the goodness-of-fit is summarized in Table 3. The coefficient of determination ($R^{2}$) exceeds 0.993 for all four coefficients, indicating excellent fitting quality.

Importing these fitted curves into TruckSim yielded more accurate simulation results. The variation trends of the aerodynamic coefficients with respect to the relative inflow angle obtained from the simulations were post-processed and used to replace the default aerodynamic coefficient functions in TruckSim. This replacement improved the accuracy of the dynamic response of the vehicle under crosswind disturbances and enhanced the data fidelity of the TruckSim simulations for the vehicle [20].

## 4. Design of an AFS System Controller Based on the LQR Algorithm

The proposed controller was developed based on vehicle-state feedback information obtained from onboard sensing systems. The measured vehicle dynamic states were continuously fed back to the LQR controller for corrective steering control. The overall design concept of the AFS controller based on the LQR algorithm is as follows:

(13) $$\dot{X}=AX+BU,$$

(14) $$J=\frac{1}{2}\int_{t_{0}}^{t_{f}}\left[X^{T}Q_{1}X+U^{T}Q_{2}U\right]dt+\frac{1}{2}X^{T}\left(t_{f}\right)Q_{0}\left(t_{f}\right),$$

where U is the control vector, X is the state variable, $Q_{0}$ is the terminal weighting matrix, $Q_{1}$ is the weighting matrix for the state vector, and $Q_{2}$ is the weighting matrix for the control vector.

A state regulator was employed to minimize the performance index. An optimal control law U was sought over the time interval [t0, tf] to drive the system from its initial state to the equilibrium state, while minimizing the performance index. The control horizon was first defined as [0, ∞]. The specific expression of the performance index is given by

(15) $$J=\frac{1}{2}\int_{0}^{\infty }\left[X^{T}Q_{1}X+U^{T}Q_{2}U\right]dt.$$

The optimal state feedback gain matrix is denoted as K, where U = −KX. From this, $K=Q_{2}^{-1}B^{T}P$ is obtained. Thus, the control design problem was reduced to solving for the feedback gain matrix K and the algebraic Riccati equation, which is expressed as follows:

(16) $$PA+A^{T}P-PBQ_{2}^{-1}B^{T}P+Q_{1}=0.$$

**Figure 12:**
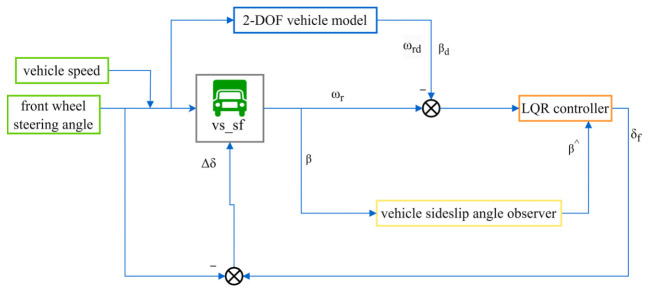
Sensor-feedback-based control framework of the AFS system.

As expressed in Equation (16), the optimal solution depends on the choices of Q_1_ and Q_2_. The AFS controller intervened when the actual yaw rate and the vehicle sideslip angle deviated significantly from the reference values. The control algorithm then computed an additional front wheel steering angle △δ and fed it to the vehicle model in TruckSim. This additional angle was added to the actual front-wheel steering angle, yielding the current front-wheel steering angle used in TruckSim. Thus, the vehicle-handling stability was improved. The overall control framework is shown in Figure 12.

As illustrated in Figure 12, the ideal 2-DOF vehicle model generates the reference yaw rate and reference vehicle sideslip angle based on the vehicle speed and steering input. The actual yaw rate and vehicle sideslip angle are obtained from the TruckSim vehicle model and represent the feedback information provided by onboard sensing systems. The state errors between the reference values and the measured values are then sent to the LQR controller. According to these errors, the controller calculates an additional steering angle, which is superimposed onto the driver steering input and applied to the front wheels. Through this closed-loop feedback mechanism, the vehicle continuously corrects its motion states and maintains handling stability under crosswind disturbances.

In practical LQR design, the weighting matrices should reflect the relative importance and physical limitations of vehicle dynamic variables. In this study, the reference yaw rate and sideslip angle bounds derived in Equation (7) were used as references when selecting the state weighting factors. Greater penalties were assigned to state variables that are closely related to vehicle stability, namely the yaw rate tracking error and sideslip angle tracking error, while the control weighting matrix was used to prevent excessive additional steering commands. Therefore, the weighting matrices were selected to achieve a balance between tracking performance and steering effort under crosswind disturbances.

### 4.1. Cost Function Design

Subsequently, a discrete LQR controller was designed. The discrete state-space representation of the system is given by

(17) $$x_{k+1}=A^{*}x_{k}+B^{*}u_{k}.$$

The system was discretized using the forward Euler method, yielding discrete-time system matrices $A^{*}=A\;*\;dt+I\;and\;B^{*}=B\;*\;dt,$ where dt denotes the discrete time step and I is the identity matrix.

The control objective is the desired state $x_{d}$, a new augmented state variable $x_{a}$ is defined, and the corresponding state-space equation is written as follows:

(18) $$x_{a|k}=\left[\begin{array}{c} x_{k}\\ x_{d|k} \end{array}\right],$$

(19) $$x_{a|k+1}=A_{a}x_{a|k}+B_{a}u_{k}.$$

The complete state-space equation is given by

(20) $$\left[\begin{array}{c} x_{k+1}\\ x_{d|k+1} \end{array}\right]=\left[\begin{array}{cc} A^{*} & 0_{2\times 2}\\ 0_{2\times 2} & I_{2\times 2} \end{array}\right]\left[\begin{array}{c} x_{k}\\ x_{d|k} \end{array}\right]+\left[\begin{array}{c} B^{*}\\ 0_{2\times 1} \end{array}\right]u_{k}.$$

The control output of the system is defined as the tracking error:

(21) $$y=e_{k}=x_{k}-x_{d|k}=Cx_{a|k},$$

where $C=\left[I_{2\times 2},-I_{2\times 2}\right]$.

The desired motion of the vehicle was tracked by driving the tracking error to zero as follows:

(22) $$J=\sum_{k=0}^{\infty }y_{k}^{T}Q_{0}y_{k}+u_{k}^{T}Ru_{k}=\sum_{k=0}^{\infty }x_{a|k}^{T}C^{T}Q_{0}Cx_{a|k}+u_{k}^{T}R_{0}u_{k}.$$

Let $C^{T}Q_{0}C=Q\;and\;R=R_{0}$. The cost function is then transformed into a state regulation problem, yielding:

(23) $$J=\sum_{k=0}^{\infty }x_{a|k}^{T}Qx_{a|k}+u_{k}^{T}Ru_{k}.\;$$

The weighting matrices of the LQR controller were determined through repeated simulation-based tuning. Different weighting combinations were evaluated in terms of yaw rate tracking accuracy, vehicle sideslip angle suppression, and steering control effort under crosswind disturbances. The final parameter set was selected because it achieved a satisfactory balance between vehicle handling stability and control input while maintaining stable closed-loop performance throughout the investigated operating conditions. Since the primary objective of this study is to validate the effectiveness of the proposed AFS-LQR framework, a systematic sensitivity analysis of the weighting matrices was not conducted. The influence of weighting-matrix selection on controller performance will be investigated in future work.

### 4.2. Discrete LQR Solution

1. Derivation of the LQR feedback gain

The discrete state-space equation of the system is given by:

(24) $$x_{\left(k+1\right)}=A^{*}x_{k}+B^{*}u_{k}.$$

First, the optimal solution of the cost function was obtained through backward recursion. For computational convenience, the cost function can be rewritten as

(25) $$J=\frac{1}{2}\sum_{k=0}^{\infty }\left[x_{k}^{T}Qx_{k}+u_{k}^{T}Ru_{k}\right].$$

(1) The cost incurred by the system from k = N to k = N is defined as

(26) $$J_{N\to N}\left(x_{N}\right)=\frac{1}{2}x_{N}^{T}Qx_{N}.$$

For notational consistency in the following derivation, $P_{0}=Q$ is defined as

(27) $$J_{N\to N}\left(x_{N}\right)=\frac{1}{2}x_{N}^{T}P_{0}x_{N}.$$

(2) Proceeding with the derivation of the cost function from k = N − 1 to k = N yields

(28) $$J_{N\to 1\to N}\left(x_{N},x_{N-1},u_{N-1}\right)=\frac{1}{2}x_{N}^{T}P_{0}x_{N}+\frac{1}{2}x_{N-1}^{T}Qx_{N-1}+\frac{1}{2}u_{N-1}^{T}Ru_{N-1}.$$

Substituting the state equation $x_{N}=A^{*}x_{N-1}+B^{*}u_{N-1}$ into Equation (28), differentiating with respect to $u_{N-1}$, and setting the derivative to zero yields

(29) $$B^{*T}P_{0}(A^{*}x_{N-1}+B^{*}u_{N-1})+Ru_{N-1}=0.$$

Solving the rewritten cost function yields the optimal control policy, which is expressed as

(30) $$u_{N-1}=-{\left(B^{*T}P_{0}B^{*}+R\right)}^{-1}B^{T}P_{0}A^{*}x_{N-1}.$$

The optimal feedback gain matrix is defined as $K_{N-1}={\left(B^{*T}P_{0}B^{*}+R\right)}^{-1}B^{T}P_{0}A^{*}$, which yields

(31) $$u_{N-1}=-K_{N-1}x_{N-1}.$$

Substituting the obtained $u_{N-1}$ into $J_{N-1\to N}(x_{N-1},u_{N-1})$, gives

(32) $$J_{N-1\to N}(x_{N-1},u_{N-1})=\frac{1}{2}x_{N-1}^{T}P_{1}x_{N-1},$$

which has the same form as the initial $J_{N\to N}$.

Here,

(33) $$P_{1}=(A^{*}-B^{*}K_{N-1}{)}^{T}P_{0}(A^{*}-B^{*}K_{N-1})+K_{N-1}^{T}RK_{N-1}+Q.$$

Continuing the calculation for $J_{N-2\to N}$ in the same manner yields:

(34) $$J_{N-2\to N}\left(x_{N-2},u_{N-2}\right)=\frac{1}{2}x_{N-2}^{T}P_{2}x_{N-2},\;$$

where

(35) $$P_{2}=(A^{*}-B^{*}K_{N-2}{)}^{T}P_{1}(A^{*}-B^{*}K_{N-2})+K_{N-2}^{T}RK_{N-2}+Q.$$

Each iteration produced an identical computational structure. Consequently, when the number of iterations was sufficiently large, the feedback gain K converged to a constant value, indicating that a steady state was attained.

2. Solution Procedure for the LQR Feedback Gain

Step 1: Initialization

(1) Set $P_{0}=Q$.
(2) Set the gain difference between successive steps to diff=inf and define the steady-state threshold, which determines whether the system has reached a steady state.
(3) Set the initial feedback gain $K_{N}=inf$.
(4) Set the maximum number of iterations to limit the iteration count.

Step 2: Iteration for k=1−max_iter

(1) Compute the feedback gain:

(36) $$K_{N-K}={\left(B^{*T}P_{k-1}B^{*}+R\right)}^{-1}B^{T}P_{k-1}A^{*}$$

(2) Update $P_{k}$:

(37) $$P_{k}=(A-BK_{N-k}{)}^{T}P_{k-1}(A-BK_{N-k})+K_{N-k}^{T}RK_{N-k}+Q$$

(3) Compute the gain difference between successive steps:

(38) $$diff=\max\left(abs\left(K_{N}-K_{N-1}\right)\right)$$

(4) Termination check:

If diff<threshold, the iteration is terminated. If the number of iterations exceeds the maximum allowed value, an error occurs.

## 5. Simulation Verification and Analysis of the AFS Controller

**Figure 13:**
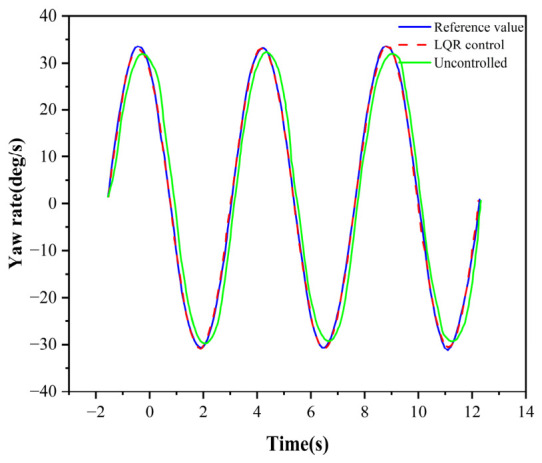
Comparison of actual and reference yaw-rate responses under sinusoidal steering.

**Figure 14:**
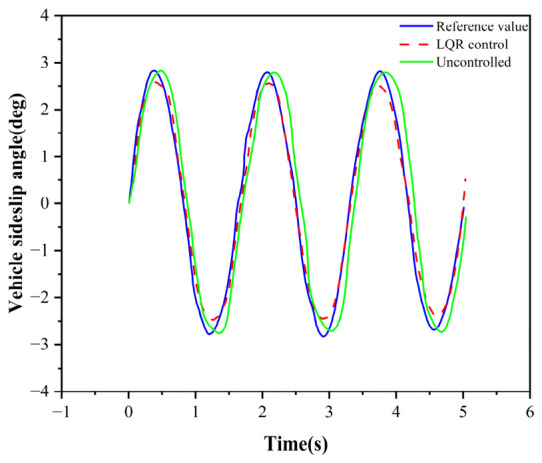
Comparison of actual and reference vehicle sideslip-angle responses under sinusoidal steering.

A continuous sinusoidal steering test was conducted to evaluate the dynamic performance of the special vehicle under crosswind disturbances. The simulation conditions were set to a vehicle speed of 80 km/h, road adhesion coefficient of 0.8, and crosswind speed of 12 m/s. The front-wheel steering angle input for this test and the detailed simulation results are shown in Figure 13 and Figure 14.

Figure 13 and Figure 14 demonstrate that the proposed AFS controller tracked the reference yaw rate and vehicle sideslip angle with high accuracy. Compared with the uncontrolled case, the vehicle under LQR control achieved considerably better path tracking and was able to follow the intended trajectory. Additionally, the actual dynamic response, specifically, the vehicle sideslip angle, consistently followed its reference with negligible error, thereby achieving stable vehicle operation under specified driving conditions.

Peak yaw-rate error denotes the absolute difference between the peak values of the actual yaw rate and the reference yaw rate.

To further quantify the controller performance, the tracking responses were evaluated using several performance indicators, as summarized in Table 4. Compared with the uncontrolled case, the proposed LQR controller reduced the peak yaw rate error from 1.21 deg/s to 0.16 deg/s, corresponding to an improvement of 86.9%. The RMS yaw rate tracking error was reduced from 4.27 deg/s to 0.63 deg/s, representing an improvement of 85.2%, while the RMS sideslip angle tracking error decreased from 0.60 deg to 0.24 deg, corresponding to an improvement of 60.0%. In addition, the maximum vehicle sideslip angle was reduced from 2.82 deg to 2.59 deg, corresponding to a reduction of 8.2%. These results further confirm the effectiveness of the proposed controller in improving vehicle handling stability under aerodynamic disturbances.

## 6. Conclusions

This study focused on a special vehicle and investigated an AFS system based on the LQR algorithm. The aim was to enable the AFS controller to improve the steering performance and help the vehicle maintain a stable body attitude when driving under external crosswind disturbances. The proposed control strategy was validated using the TruckSim simulation platform.

The AFS control strategy developed in this study, built on the LQR algorithm, enhanced the driving safety of the vehicle. This allowed the vehicle to achieve better steering performance in scenarios in which conventional steering systems fall short. Under this control strategy, the vehicle sideslip angle and yaw rate were forced to track the reference values. The controller output an additional front-wheel steering angle, which assisted the vehicle in following its intended path more accurately. The validation results demonstrate that the steering performance was significantly improved with the LQR algorithm, and the vehicle maintained a stable body attitude under crosswind disturbances. Thus, the algorithm improved both the handling stability and driving safety of the vehicle. Furthermore, the proposed sensor-feedback-based AFS framework demonstrates the application potential of vehicle dynamic sensing technologies in improving handling stability under aerodynamic disturbances.

Although the simulation results demonstrate the effectiveness of the proposed AFS-LQR controller, the present study is limited to numerical validation using the TruckSim/Simulink co-simulation platform. Experimental validation and comparative studies with other advanced control strategies, such as PID-based and MPC-based controllers, were not included in the current work. Therefore, the practical implementation performance and relative advantages of the proposed controller require further investigation.

Future work will explore integrated chassis control that combines the proposed AFS-LQR strategy with advanced suspension systems [21] to further enhance vehicle stability under complex driving conditions, including crosswind and road irregularities. In addition, more comprehensive validation scenarios—such as double-lane-change maneuvers, step steering inputs, varying crosswind intensities, different road adhesion coefficients, and multiple vehicle speeds—will be conducted to further demonstrate the robustness of the proposed controller.

## Figures and Tables

**Table 1:** Main parameters of the special vehicle.

Parameter	Unit	Value
Sprung mass	kg	6500
Curb mass	kg	7800
Distance from center of mass to front axle	mm	1400
Distance from center of mass to ground	mm	1250
Roll moment of inertia	kg/m^2^	3332.3
Pitch moment of inertia	kg/m^2^	51,653.9
Yaw moment of inertia	kg/m^2^	50,778.2

**Table 2:** The main parameters of tires of each axle.

Parameter Name	Unit	Turning Shaft	Drive Shaft
Effective Rolling Radius	mm	502	505
No-load radius	mm	518	516
Width	mm	290	260

**Table 3:** Goodness of fit.

Aerodynamic Coefficient	RMSE	SSE	$R^{2}$
$C_{d}$	0.02936	0.004962	0.9931
$C_{s}$	0.01453	0.013625	0.9968
$C_{l}$	0.09084	0.045697	0.9937
$C_{ym}$	0.02327	0.002698	0.9968

**Table 4:** Quantitative Performance Evaluation.

Metric	Uncontrolled	LQR Control	Improvement
Peak yaw-rate error (deg/s)	1.21	0.16	86.9%
RMS yaw-rate tracking error (deg/s)	4.27	0.63	85.2%
Maximum sideslip angle (deg)	2.82	2.59	8.2%
RMS sideslip-angle tracking error (deg)	0.60	0.24	60.0%

## Data Availability

The data used in this study are not publicly available as they are internal datasets of the research group. The data may be made available from the corresponding author upon reasonable request.

## Abbreviations

The following abbreviations are used in this manuscript:

2-DOF	Two-degree-of-freedom
AFS	Active front steering
LQR	Linear quadratic regulator
LTVMPC	Linear time-varying model predictive control
